# Ultrasound Imaging in Radiation Therapy: From Interfractional to Intrafractional Guidance

**DOI:** 10.7759/cureus.280

**Published:** 2015-06-20

**Authors:** Craig Western, Dimitre Hristov, Jeffrey Schlosser

**Affiliations:** 1 Department of Mechanical Engineering, Stanford University; 2 Department of Radiation Oncology, Stanford University; 3 Founder, SoniTrack Systems

**Keywords:** ultrasound, robotics, image guidance, image-guided therapy, real-time system, treatment planning, liver cancer, prostate cancer, image-guided radiation therapy

## Abstract

External beam radiation therapy (EBRT) is included in the treatment regimen of the majority of cancer patients. With the proliferation of hypofractionated radiotherapy treatment regimens, such as stereotactic body radiation therapy (SBRT), interfractional and intrafractional imaging technologies are becoming increasingly critical to ensure safe and effective treatment delivery. Ultrasound (US)-based image guidance systems offer real-time, markerless, volumetric imaging with excellent soft tissue contrast, overcoming the limitations of traditional X-ray or computed tomography (CT)-based guidance for abdominal and pelvic cancer sites, such as the liver and prostate. Interfractional US guidance systems have been commercially adopted for patient positioning but suffer from systematic positioning errors induced by probe pressure. More recently, several research groups have introduced concepts for intrafractional US guidance systems leveraging robotic probe placement technology and real-time soft tissue tracking software. This paper reviews various commercial and research-level US guidance systems used in radiation therapy, with an emphasis on hardware and software technologies that enable the deployment of US imaging within the radiotherapy environment and workflow. Previously unpublished material on tissue tracking systems and robotic probe manipulators under development by our group is also included.

## Introduction and background

External beam radiation therapy (EBRT) is used to treat > 60% of all cancer patients. Potent radiation doses with minimal treatment margins must be delivered in EBRT to maximize local tumor control and minimize toxicity to surrounding healthy tissue [1-3], but internal anatomy motion and deformation pose a fundamental threat to realizing these objectives. With the proliferation of hypofractionated radiotherapy treatment regimens, such as stereotactic body radiation therapy (SBRT), interfractional and intrafractional imaging technologies are becoming increasingly critical to ensure safe and effective treatment delivery [3-12].

The current gold standard for tumor motion management is X-ray imaging of implanted fiducial markers [13-16]. However, limitations, including marker migration [17-20], additional ionizing radiation exposure, implantation obstacles and morbidity [18-22], and the lack of volumetric information [17, 23-24], limit its utility. Cone-beam computed tomography (CBCT) produces a volumetric image of the patient’s anatomy prior to treatment but cannot be used to obtain intrafractional motion information [25]. Electromagnetic marker tracking enables real-time radiation-free motion tracking [26-28], but markers produce severe artifacts when used with computed tomography (CT) and magnetic resonance imaging (MRI) and do not yield volumetric images. Recent attempts at real-time, volumetric, markerless tracking for radiotherapy include using MRI and positron emission tomography (PET), but these approaches require the construction of new therapy machines with integrated imaging capabilities [29-31].

Ultrasound (US) offers an attractive alternative to the previously described approaches for imaging EBRT targets outside the skull and lung. Commonly used for image guidance in brachytherapy (robotic [32] and non-robotic [33]), biopsy [34], and other medical procedures, US imaging systems offer nonionizing, real-time volumetric imaging with excellent soft-tissue contrast. This paper describes various commercial and research-level US EBRT guidance systems, with specific emphasis on hardware and software technologies that enable the deployment of US imaging within the EBRT environment and workflow. Section 1 describes commercial interfractional US imaging systems, while Sections 2, 3, and 4 outline the various technical aspects of newer intrafractional US guidance systems, including static and robotic probe fixtures (Section 2), automatic tissue tracking software (Section 3), and treatment planning and spatial probe tracking sub-systems (Section 4). Previously unpublished material on tissue tracking systems and robotic probe manipulators under development by our group is included in Sections 2 and 3.

## Review

### 1.  Interfractional US guidance

The first applications of US in radiotherapy utilized US to image anatomy prior to treatment and inform patient placement relative to the linear accelerator (LINAC).

1.1     BAT and SonArray Systems

**Figure 1:**
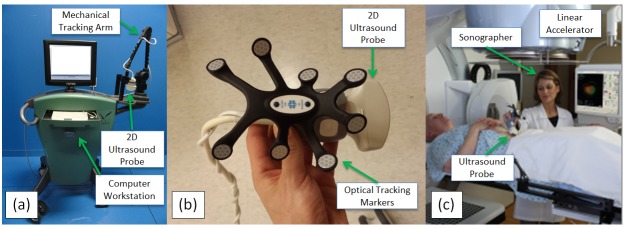
Interfractional US guidance systems. (a) NOMOS B-mode Acquisition and Targeting (BAT), courtesy of Medicka Medical LLC. (b) Elekta Clarity probe. (c) Clarity probe in use, courtesy of Elekta, Inc.

The first widely adopted US systems for interfractional imaging were the B-Mode Acquisition and Targeting (BAT) system (NOMOS Corp., Cranberry Township, PA) (Figure 1A), introduced in the United States markets in the late 1990s, and the SonArray system (Varian Medical Systems, Palo Alto, CA), introduced in the years following [35]. These systems use a 2D diagnostic US imaging system (refer to Section 4.1) and an optical or mechanical means (refer to Section 4.2) for tracking the position of the US probe with respect to the LINAC. In the BAT system, transabdominal US is used to image the target region in two near-orthogonal planes [36]. In the SonArray system, a sonographer sweeps the US probe across the target anatomy, capturing a series of 2D slices that are reconstructed into a 3D volume based on the relative slice positions. In both systems, the patient is positioned with respect to the LINAC prior to beam delivery by matching the planning CT volume to US images spatially localized in the LINAC frame [36-39].

When the BAT system arrived on the market, US was the only method for obtaining soft-tissue interfractional images (CBCT was not introduced until 1996 in Europe and 2001 in the United States) [40]. Little, et al. [38] found that without orthogonal plane US imaging, prostate motion would have caused the target to move outside of the planning target volume (PTV) in 23.3% to 41.8% of cases, concluding also that prostate organ motion dominates setup error. Studies of patients with prostate cancer by Lattanzi, et al. [36] demonstrated that alignment errors between a baseline CT and US after initial positioning often exceeded 5 mm using the BAT system, with maximum ranges of -26.8 to 33.8 mm in the anteroposterior (AP) direction, -10.2 to 30.9 mm in the mediolateral (ML) direction, and -24.6 to 9.0 mm in the superoinferior (SI) dimension. Boda-Heggemann, et al. [39] found that the BAT system improved positioning accuracy when compared to positioning using bony anatomy in CBCT scans and significantly improved positioning accuracy over skin marks (with magnitudes of residual error relative to positioning based on skin marks between 2.1 and 5.2 mm).

Several studies have compared BAT and SonArray positioning accuracy with X-ray fiducial marker positioning. Langen, et al. [41] showed that prostate alignment using the BAT system systematically differs from alignment using radiographic fiducial marker imaging in the SI (2.7 ± 3.9 mm) and ML (1.6 ± 3.1 mm) directions, but differences were minimal in the AP direction (0.2 ± 3.7 mm). Van den Heuvel, et al. [42] found that position shifts suggested by the BAT system were similar to fiducial marker shifts in the AP and ML directions but differed significantly in the SI direction, suspecting a systematic error in their BAT positioning system. Peignaux, et al. [43] found significant differences in SonArray vs. X-ray fiducial marker positioning along the SI and AP axes. Scarbrough, et al. [44] found a three-dimensional distance discrepancy between the two methods of 8.8 mm (significantly > 5 mm), concluding that larger target volume margins are necessary for SonArray (approximately 9 mm) as compared with kV X-ray (approximately 3 mm) in intrafractional imaging applications.

Although initial studies of the BAT and SonArray systems demonstrated the promise of improved soft tissue-based prostate positioning, cross-examination with X-ray fiducial imaging revealed systematic biases. Reliance on intermodality matching of US and CT image information is partially responsible for these systematic errors. For example, prostate volumes derived from CT scans are consistently larger than those derived from US images because of differing physical image contrast mechanisms and the inability of CT to differentiate structures well at low contrast [35]. Molloy, et al. [45] estimated prostate size differences to be up to 9 mm in the ML direction and 3 mm in the AP direction. Cury, et al. [46] showed systematic differences in intermodality and intramodality positioning, finding significant mean differences of 0.9 ± 3.3 mm in the ML and 6.0 ± 5.1 mm in the SI directions between the two techniques. Because a comparison of intramodality US imaging and CT scans showed no significant differences in any direction, it was concluded that intramodality-based positioning is more accurate than intermodality positioning. Therefore, intramodality image matching has been recommended to minimize patient positioning error in US-guided radiotherapy [35].

1.2     Clarity System

The Clarity System (Figure 1B-C) (Elekta AB, Stockholm, Sweden) leverages intramodality image matching instead of the intermodality matching technique used by BAT and SonArray. Intramodality matching is achieved by capturing a US volume directly before or after the planning CT scan. By tracking the precise position and orientation of the US probe with respect to the CT scanner, the 3D US volume can be reconstructed in the reference frame of the CT. Directly prior to treatment, a new 3D US volume is captured in the LINAC room and reconstructed with respect to the LINAC reference frame. To position the patient, the US volumes collected during planning and prior to treatment are matched and used to determine patient anatomy offset relative to the desired planning position [47]. Since intramodality image matching has been shown to improve accuracy over intermodality matching as described in Section 1.1, the Clarity system should theoretically position patients more accurately than the BAT system or SonArray system.

Gurp, et al. [48] demonstrated feasibility of US-based image guidance in SBRT of liver lesions using the Clarity system, finding that the variability of image segmentation in scanned images was 4 mm (one standard deviation), and could be reduced by 1.7 mm in the SI direction using active breathing control. To evaluate the error in the Clarity system applied to breast imaging, Wong, et al. [49] compared CT scans of breast cancer patients taken just before radiation to Clarity US images taken at corresponding times. The difference between CT and US images of the tumor bed centroid position averaged 0.1 ± 2.8 mm, -0.2 ± 4.0 mm, and 0.4 ± 3.7 mm in the AP, LR, and SI directions, respectively, which was deemed clinically insignificant. Robinson, et al. [50] found the prostate positioning error between Clarity scans and CT scans compared to a reference scan to be significant, observing a discrepancy of 5 mm or more between CT and US localizations in > 80% of cases despite expert re-analysis of data.

While the Clarity System leverages intramodality image matching, an improvement relative to the intermodality matching used by the BAT and SonArray systems, conflicting reports of Clarity’s positioning accuracy bring to light the shared shortcomings of interfractional US image guidance systems:

1.  Pressure applied by the US transducer on the patient’s body can cause anatomy deformations and displacements of varying magnitude, depending on the properties and depth of the treatment target. Since pressure applied during pre-treatment imaging is not present during beam delivery, systematic positioning errors are common [51-53].

2. The quality and consistency of freehand US-based patient alignment is significantly dependent on the operator and level of training [41, 54].

3. The Clarity and BAT systems rely on freehand probe manipulation, which would pose an unacceptable hazard to the probe operator if performed during treatment delivery. Thus, no imaging is available during beam delivery when accurate target tracking is most critical.

### 2.  Intrafractional US guidance: Image acquisition devices

Intrafractional US guidance systems are the next step in the evolution of US imaging for radiotherapy, providing real-time, volumetric, markerless target tracking concurrent with beam delivery in packages that integrate with existing LINACs. The foundation of an intrafractional US guidance system is a hardware device to maintain the US probe in imaging position during therapy while the sonographer is outside the treatment room. The device must hold the probe in a way that maintains the therapy target within the US imaging field of view throughout treatment while minimizing possible interference with the LINAC, the patient’s body, and treatment beams. This section describes several intrafractional US image acquisition devices currently in clinical use or in development.

2.1     Static Devices

**Figure 2:**
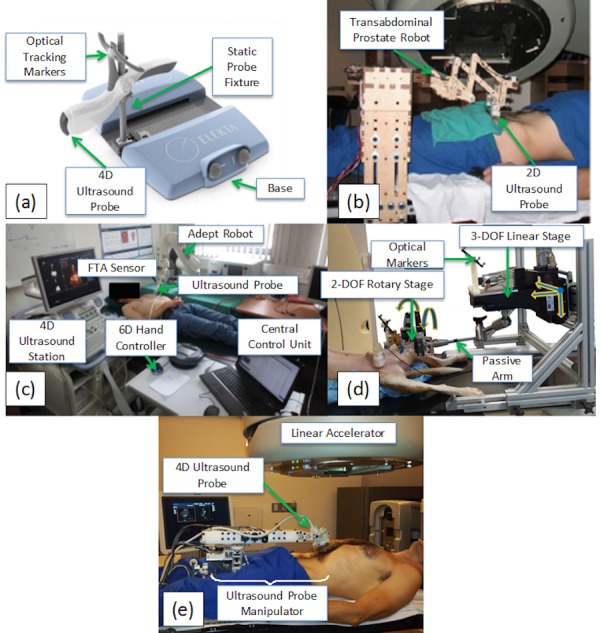
Devices to support intrafractional US imaging. (a) Clarity Autoscan system, reproduced from Lachaine and Falco[47]. (b) Stanford prostate imaging robot, reproduced from Schlosser et al.[58] (c) University of Lubeck robot, adapted from Kuhlemann[59]. (d) Johns Hopkins robot, adapted from Sen et al.[61] (e) Stanford abdominal imaging robot.

The first US guidance system capable of intrafractional imaging was the Clarity Autoscan System (Elekta AB, Stockholm, Sweden), shown in Figure 2A. The Clarity Autoscan builds upon the original Clarity System (Section 1.2) by replacing the 2D US imaging system with a mechanical “wobbler” 3D/4D US imaging system (refer to Section 4.1) and adding a hardware fixture for hands-free transperineal prostate imaging. The hardware device is a simple, manually-operated 5-degree of freedom (DOF) fixture that is mounted to a plate on the treatment couch between the patient’s legs. The sonographer uses the fixture to lock the US probe into position after the initial transperineal imaging position is found, thus freeing the sonographer to exit the treatment room and deliver radiotherapy while the US probe remains in imaging position. The 3D/4D probe enables volumetric US images to be automatically captured without physically moving the US probe head. The transperineal imaging setup is advantageous because the geometry of the fixture and probe keeps all system hardware out of the normal delivery plane for C-arm LINACs, therefore avoiding guidance system hardware interference with the radiation treatment process and enabling simultaneous US/CT imaging during the radiotherapy planning phase. Clinical studies of the system are underway to characterize the performance of the Clarity Autoscan for intrafractional monitoring.

2.2     Robotic Devices

This section describes four actively-controlled (robotic) devices under development by academic institutions for maintaining probe positioning against the patient during beam delivery. Robotic ultrasound systems have been investigated in the past for diagnostic imaging purposes [55-57], but such systems are inadequate for image guidance in radiotherapy because the kinematics, workspace, and design features do not comply with the geometric and workflow constraints of the radiotherapy environment.

2.2.1     Stanford Prostate Imaging Robot

Schlosser, et. al. [58] were the first to demonstrate the feasibility of robotic intrafractional US imaging in the context of radiotherapy guidance. As a proof-of-concept, they developed and evaluated a robotic device designed for controlling 2D transabdominal US imaging of the prostate, shown in Figure 2B. The device has two active degrees of freedom for controlling probe pitch (tilt in the superior/inferior direction) and pressure against the abdomen, and three passive, mechanically locking degrees of freedom for manually setting probe position prior to the procedure. The active degrees of freedom can be remotely controlled during beam delivery using a joystick with force feedback known as a haptic device. With the telerobotic system, the authors demonstrated that gantry collisions are avoidable, stable remotely-controlled prostate imaging is achievable in healthy human subjects over 10-minute time periods, and robotic performance is not degraded during operation of a 15 MV radiation beam.

2.2.2     University of Lubeck Robotic Platform

The University of Lubeck has developed a robotic US probe positioning system for CyberKnife radiotherapy using an off-the-shelf robotic arm (Viper S850, Adept Technology, Inc.) shown in Figure 2C [59]. The robot has six actively-controlled degrees of freedom (DOF), enabling probe placement on nearly any part of the patient’s body. User commands controlling robot pose are input using a 6-DOF hand controller/computer interface and sent via TCP/ IP protocol to the control software [60]. Continuous high-quality imaging was confirmed by showing that image structure/entropy stayed above the threshold for continuous target tracking of heart volumes at least 95% of the time in three healthy human subjects over 30-minute time intervals [59]. An updated version of the system uses a KUKA (Augsburg, Germany) 7-axis lightweight robot with integrated force control. In addition to manual 6-DOF positioning, force- and image-based positioning strategies have been developed.

2.2.3     Johns Hopkins Abdominal Imaging Robot

Sen, et al. [61] have developed a custom-designed robotic manipulator, shown in Figure 2D, for US-guided radiotherapy with particular focus on overcoming inconsistencies in tumor localization between the planning and treatment phases due to tissue deformation induced by probe pressure. The design incorporates a six-axis force/torque sensor for force feedback, five active degrees of freedom (three translational and two rotational—probe spin is not actively regulated), seven passive degrees of freedom, and optical robotic position tracking. In the CT planning phase, a model probe containing no metallic components is positioned against the abdomen to cause deformation similar to that which occurs during treatment. During the treatment setup phase, a virtual spring system helps the ultrasonographer manually move the probe to a position similar to that recorded during planning, inducing repeatable deformation and repeatable force. Virtual springs consist of motors in the robotic joints that exert force toward the position recorded during planning. Flexibility in the virtual springs accommodates any changes in position that the sonographer deems necessary. Treatment planning is carried out with the model probe, which is penetrable by X-rays, held in position, such that a CT scan can be conducted. In *ex vivo* experiments using a bovine liver fixed in gelatin with implanted fiducials, the system demonstrated repeatable arm placement with minimal effect on displacement of the fiducials, yielding (after six repeated arm placements) a mean absolute difference between fiducial displacements of 0.4 ± 0.4 mm in US images and 0.3 ± 0.2 mm in CT images acquired with the model probe [62]. Later *in vivo* experiments conducted on a dog demonstrated mean 3D reproducibility of 0.6 to 0.7 mm, 0.3 to 0.6 mm, and 1.1 to 1.6 mm for the prostate, liver, and pancreas, respectively, under position control and controlled ventilation [53]. Force control proved less reproducible, however, indicating that position control rather than force control should be used for robotic substitution of real and model probes. Results indicated that the system shows promise for monitoring real-time organ motion, particularly under conditions of minimal probe pressure.

2.2.4     Stanford Abdominal Imaging Robot

Our research group at Stanford, in collaboration with SoniTrack Systems, Inc., has developed a second-generation custom-designed robotic device based on learnings from our first proof-of-concept prototype (Section 2.2.1), shown in Figure 2E. The goal of the research effort is to produce a simple, compact, human-safe robotic design that enables 3D/4D US imaging of any abdominal radiotherapy target, actively controls probe force, allows rapid and repeatable positioning of the US probe, and eliminates metal in areas exposed to CT/therapy radiation. The resulting design has a single active DOF to control probe pressure against the patient, five passive, electronically locking probe positioning DOFs, and three passive, manually actuated positioning DOFs [63]. The active DOF is controlled with a series elastic force controller that has the following advantages: (1) easy back driveability in case of power failure; (2) elimination of the need for a metallic force sensor near the US probe; and (3) inherent safety when used to control contact with human subjects. Metallic components of the robotic arm in the CT/therapy field are eliminated by coupling the 3-DOF robot wrist with remotely located sensors and actuators via a mechanical cable drive system. The 9-DOF robot design enables easy access to any abdominal target on the patient’s body while accommodating a wide range of patient body shapes and avoiding potential collisions with the rotating LINAC. The robot has demonstrated successful imaging over extended time periods on the prostate, pancreas, liver, and kidneys of healthy volunteers under informed consent.

2.2.5     Comparison

Table 1 compares the previously described static and robotic intrafractional US image acquisition systems. While static systems, such as the Clarity Autoscan, currently represent state-of-the-art clinically deployed US guidance for radiation therapy, robotic systems still in development can overcome limitations fundamental to static assemblies. While static fixtures for transperineal imaging do not interfere with radiation beams and eliminate CT scanning artifacts within the anatomy of interest, transperineal imaging is limited to the prostate as other major radiotherapy target organs cannot be reached. The Stanford prostate robot and the Lubeck robot both improve upon this by adding force control, but because of incompatibility with CT, both are unable to maintain consistent probe force between the planning and treatment phases. The Johns Hopkins robot is able to reproduce anatomical positioning during CT and radiotherapy delivery using 5-DOF actuated robotic position and force control. The Stanford abdominal robot achieves CT compatibility, continuous force control, and natural US probe positioning in a lightweight, low-cost, human-safe package.

**Table 1:** Comparison of intrafractional US image acquisition systems used in radiotherapy.

	Clarity Autoscan[47]	Stanford prostate[58]	Johns Hopkins[61]	Lubeck[59]	Stanford Abdominal[63]
For use with	3D/4D US	2D US	3D/4D US	3D/4D US	3D/4D US
Active DOF	0	2	5	6/7	1
Passive DOF	5	3	7	0	8
Probe force control	None	1 DOF	5 DOF	6 DOF	1 DOF
CT/beam compatible	Yes	No	Yes	No	Yes
Remote control interface	N/A	Haptic control	Computer interface	6D hand controller/ Computer interface	Computer interface
Organs tested	Human subjects: prostate	Human subjects: prostate, liver	Canine: Prostate, Liver, Pancreas	Human subjects: Heart, Liver	Human subjects: Liver, kidney, prostate, pancreas
Organs compatible	Prostate (transperineal)	Prostate (transabdominal), liver (in some cases)	Abdominal, pelvic	Abdominal, pelvic, thoracic	Abdominal, pelvic

### 3.  Intrafractional US guidance: Automatic tissue tracking

To enable intrafractional treatment intervention, US images collected during beam delivery must be processed in real-time to extract soft tissue motion information. Three-dimensional/four-dimensional US imaging for intrafractional guidance maximizes information content in images and enables true 3D motion tracking but suffers from slower frame rates and longer image processing times when compared with 2D US imaging. While tissue tracking algorithms are employed in a range of medical applications, this section reviews methods implemented on 2D and 3D/4D US image streams for the specific purpose of intrafractional radiotherapy guidance.

3.1     2D Tissue Tracking

**Figure 3:**
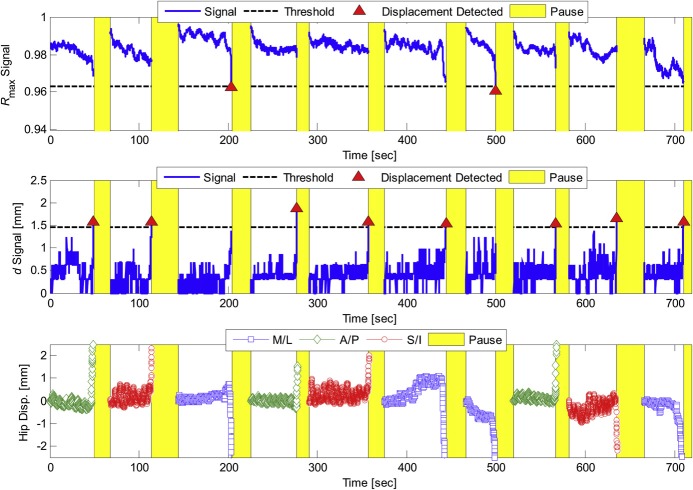
Tissue displacement parameters (TDPs) during 12-min test session, reproduced from Schlosser et al. Tissue displacement parameters (TDPs) during 12-min test session, reproduced from Schlosser et al.[64] Triangles indicate which parameter detected the displacement. Shaded regions indicate the period after the detection in which tissue monitoring was paused, a new template window was selected, and the TDPs were reset. The bottom graph shows the position of the external marker on the volunteer’s hip.

To demonstrate early feasibility of 2D US in monitoring intrafractional soft-tissue displacements of the prostate, Schlosser, et al. [64] developed a method using two tissue displacement parameters (TDPs) derived from the normalized cross correlation similarity measure that characterized in-plane and out-of-plane displacement of the target volume in real time relative to a reference position. The method, used in conjunction with the robotic device described in Section 2.2.1, successfully detected prostate displacements in healthy human subjects before they exceeded 2.3, 2.5, and 2.8 mm in the AP, SI, and ML directions, respectively, at the 95% confidence level, with a total system latency averaging 173 ms. False positives did not exceed 1.5 events over 10 minutes of continuous imaging. The authors performed an online demonstration of the system in which a healthy human subject was asked to physically move his hips at certain time intervals, causing a displacement of the prostate relative to a “world” reference frame. Hip displacements were monitored using an external marker on the volunteer’s hip and with the 2D US-based TDPs. The TDPs detected 10 out of 10 prostate displacements and registered zero false positives over the 12-minute online test. Results of the test are illustrated in Figure 3.

A similar demonstration of feasibility was carried out by Schlosser, et al. [65] using 2D US for monitoring the motion of the liver in healthy human subjects under informed consent. US image streams were acquired remotely in volunteers for 60-120 seconds using the robotic device described in Section 2.2.1. Concurrently, the position of an external infrared skin marker fixed to each subject’s abdomen was tracked. Within each image stream, the displacement of two separate liver features was monitored using normalized cross correlation, one serving as a baseline “target” and the other one as an internal target surrogate. Two models were fitted and used to predict target motion. The first one used the displacement of the external marker surrogate as an input signal. The second one used the displacement of the internal US surrogate feature as an input. Discrepancies between the measured target positions and those predicted by the models were quantified. In a separate analysis, the Pearson correlation coefficient and phase difference between the surrogate signals and the target signal were examined as a function of time.

**Figure 4:**
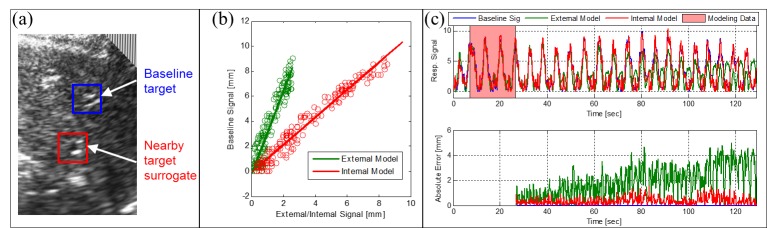
Respiratory analysis for the liver. (a) Baseline blood vessel and nearby target. (b) Linear predictive models. (c) Respiratory signals and model errors. Reproduced from Schlosser[63]

Errors based on the external surrogate model was larger than 2.0 mm on average, at times exceeding 4.0 mm while the mean error was less than 1.0 mm using the internal US surrogate model. Pearson correlation coefficient averaged 0.83 between external surrogate motion and target motion, in contrast to 0.97 between internal surrogate and target. The study thus demonstrated superior tracking of target motion using US to monitor the displacement of an internal feature when compared with tracking of an external surrogate. Results are illustrated in Figure 4.

3.2     3D/4D Tissue Tracking

Harris, et al. [66] assessed tracking accuracy and precision of real-time 4D US using a mechanically swept probe on both a tissue-mimicking phantom and on liver motion of healthy volunteers *in vivo*. Using a 3D cross correlation-based tracking algorithm, results found non-incremental tracking (comparing each volume with the first volume) to be superior to incremental tracking (comparing each volume to the next) and good agreement between *in vivo* cases tracked automatically and manually, with 1.7 mm mean absolute distance of the difference between the two methods. Subsequent studies [67] conducted on liver motion *in vivo* examined the effect of volumetric imaging rates using a 4D matrix array US probe, finding that lower volume rates (2-12 Hz) resulted in root mean squared deviation (RMSD) values of 2-6 mm relative to the highest rates (24 Hz). In a third study, the accuracy of a mechanically-swept 4D US probe for transperineal monitoring of prostate motion was evaluated using a US phantom undergoing prescribed motion [68]. The system tracked SI and AP motion to ≤ 0.81 mm RMS error at a 1.7 Hz volume rate as compared with 0.74 mm for the CyberKnife system to which volumetric US was being compared. The error was higher in the ML direction (elevational sweep direction for the US probe) but could be reduced to ≤ 2.0 mm using a correlational threshold.

The Clarity Autoscan System monitors intrafractional prostate motion by continuously gathering swept 2D slices of the region of interest in a cylindrical coordinate frame [47]. Rather than wait for a full 3D US sweep, 2D slices are registered in succession on each partially updated image using normalized cross correlation. The registration algorithm calculates a correlation score for each iteration, and if the correlation is below a threshold chosen based on training data, a displacement is suspected and the user is alerted to verify the registration. Lachaine and Falco [47] showed that the accuracy of the Clarity system is -0.2 ± 0.2 mm, 0.2 ± 0.4 mm, and 0.0 ± 0.2 mm in the AP, LR, and SI directions, respectively, by comparing Clarity tracking of a phantom with motion patterns fed to a robotic stage. In a similar study, Abramowitz, et al. [69] showed 95% of maximum distance variation between Clarity measurements and optical control measurements of a robotically controlled probe to be less than 1.3 mm, with the majority less than 1 mm. In both studies, target geometry and motion were informed by prostate motion profiles.

In the robotic system developed by Bruder, et al. [70], template matching algorithms using the sum of squared differences (SSD) are employed for the purpose of tissue tracking. Similar to previously described methods, the process involves the comparison between the current US volume and a template – a volume previously captured at a specific position. Unlike previously described rigid tracking algorithms, Bruder accounts for target deformation by developing a multi-template matching algorithm in which the current US volume is compared with a number of templates that represent snapshots of the patient’s anatomy across a range of deformations – for example, through a single breathing cycle. The algorithm is successfully employed in the University of Lubeck robotic system (Section 2.2.2) with 15 ms of processing time in practice.

Kubota, et al. [71] have proposed a 3D tissue tracking algorithm for monitoring organs affected by respiratory motion. The algorithm first required manually identifying a region of interest that enveloped the target. A direction of maximum displacement due to respiratory motion was then identified prior to tracking by averaging images acquired at maximum inspiration and expiration. In tracking target motion in real-time, a Pyramidical Lucas-Kanade method was used to associate a large number of feature points in each frame with points in the previous frame, then moving the region of interest in a direction associated with the motion of these points. In order to adjust for deformations and stacked error over time, error correction was applied by comparing images at maximum inspiration and expiration over multiple cycles and updating, if necessary, under the assumption that target position in the image of maximum inspiration was constant between cycles. The method was validated by tracking the gallbladder in one subject and a liver vein in a second subject. The proposed algorithm was compared with a template matching algorithm and a second algorithm involving feature point tracking without error correction. The proposed algorithm outperformed both alternatives, allowing longer tracking times (up to 5 minutes) and consistent tracking through organ deformation and changes in cross-sectional position. Average tracking accuracy was 1.54 ± 0.9 mm, with accuracy defined as deviation from the center of the region of interest and the center of the target organ designated by an experienced medical doctor. Computation time was 8 ms per frame for 2000 frames processed.

Table 2 reviews the previously discussed tissue tracking systems.

**Table 2:** Comparison of US tissue tracking systems used in radiotherapy.

Study	Method	2D/3D	Temporal Characteristics	Accuracy	Organs Tested	Evaluated In Vivo or Phantom
Schlosser et al. 2012 [64]	Tissue displacement parameters based on 2D normalized cross-correlation	2D	173 ms system latency; 9 Hz frame rate	< 2.8 mm at 95% confidence	Prostate	In vivo
Schlosser et al. 2011 [65]	2D normalized cross-correlation	2D	173 ms system latency; 9 Hz frame rate	< 1 mm mean	Liver	In vivo
Harris et al. 2010 [66]	Non-incremental 3D cross correlation	3D	Not reported	1.7 mm in vivo	Liver	In vivo, phantom
O’Shea et al. 2014 [68]	3D cross-correlation with block matching	3D	1.7 Hz frame rate	≤0.81 mm in phantom case, SI and AP directions	N/A	Phantom
Clarity Autoscan [47, 69]	2D/3D normalized cross-correlation	2D/ 3D	0.5 second sweep time or greater depending on resolution	0.2 ± 0.4 mm worst-case for AP, ML, and SI directions (Lachaine); 1.3 mm (Abramowitz)	N/A	Phantom
Bruder et al. 2009 [70]	SSD and multi- template matching	3D	50 ms system latency; 20 Hz frame rate	Not reported	Heart, liver	In vivo
Kubota et al. 2014 [71]	Feature point tracking with error correction algorithm	3D	8 ms image processing time	1.54 ± 0.9 mm	Gallbladder, liver vein	In vivo

### 4.  Additional considerations for US-guided EBRT

4.1     US Imaging Probes

In US imaging, acoustic waves are sent and received by a US probe coupled to a scanned volume of tissue. Variation in physical properties of the scanned volume causes a proportion of the outgoing acoustic energy to be reflected back to the probe. By recording the amplitude and timing of reflections and assuming a particular speed of sound, an image of the scanned volume can be reconstructed. Two-dimensional US probes propagate acoustic waves in a single plane within the patient’s body, either using a single mechanically-steered transducer element, or more commonly, using a linear or curvilinear array of transducer elements [72]. In 3D/4D US probes, waves are propagated throughout a 3D volume of tissue (4D imaging refers to continuous real-time 3D imaging). A 3D/4D “wobbler” probe mechanically sweeps a linear or curvilinear array of transducers to collect multiple 2D planes within a volume region of interest [73], while a matrix array 3D/4D probe uses a 2D matrix of transducer elements to electronically steer an acoustic beam within a 3D volume [74].

Two-dimensional US probes are used in the BAT, SonArray, and Clarity interfractional patient positioning systems, along with a position sensor (Section 4.2) to facilitate the reconstruction of individual 2D slices into a 3D volume (Section 1.1). Two-dimensional US probes can also be useful in intrafractional monitoring systems where the target anatomy is relatively stationary [47, 64] or when motion can be captured within a single plane [65]. Three-dimensional/four-dimensional probes are superior to 2D probes for tracking generalized 3D anatomy motion in intrafractional applications (Section 3.2).

4.2     US Probe Tracking Techniques

In order to image and track patient anatomy with respect to the treatment beam, US image information must be transformed to the reference frame of the LINAC. As part of this process, the position of the US probe with respect to the LINAC must be known. Potential techniques available to determine and track probe position in the frame of the linear accelerator include optical tracking, electromagnetic (EM) tracking, mechanical tracking, and X-ray tracking.

Optical tracking uses a system of cameras fixed in the LINAC frame and a set of tracked markers attached to the US probe. Spatial and temporal resolution is high and, for this reason, most US guidance systems use this modality [47, 58, 61], but optical tracking systems are limited by line-of-sight requirements between the camera and markers. EM tracking uses an EM emitter on the probe and a detector fixed in the LINAC room. EM tracking does not necessarily require a clear optical line of sight between emitter and detector but is very susceptible to noise caused by metallic objects in the operating vicinity. Devices used to hold the US probe in place during intrafractional tracking (Section 2) can also be leveraged for tracking the probe. If the static or robotic device has sensors to measure each joint angle, the angles in combination with knowledge of the device geometry (forward kinematics) can be used to locate the probe in space. This tracking method is independent of the surrounding environment, but high accuracy can only be achieved by using very precise device manufacturing techniques and rigid materials. Our group is currently investigating the performance and tradeoffs between optical, EM, and mechanical tracking in the radiotherapy environment.

Bruder, et al. [75] investigated an approach to US probe localization utilizing the stereo X-ray cameras of the CyberKnife and X-ray markers attached to the probe (Accuray, Inc., Sunnyvale, CA). After a non-orthogonal stereo camera calibration using X-ray phantoms, various marker geometries were positioned using a 6-axis robotic arm, localized in 6-DOF using algorithms developed for marker localization, and compared with results from the CyberKnife onboard system. Mean translational error for the newly developed software package was 0.218 mm and rotational error was 0.076 degrees. Although spatial accuracy is high, this probe localization method would not be appropriate for continuously tracking the respiratory-induced probe motion as X-ray exposure practically limits the temporal resolution.

Table 3 provides a summary of the previously discussed probe tracking techniques.

**Table 3:** Comparison of US probe tracking techniques available in radiotherapy.

	Optical	Electromagnetic	Mechanical	X-ray
Typical spatial resolution	0.2-0.4 mm	0.5-0.8 mm	0.1-5.0 mm	0.2 mm
Typical acquisition rate	20-60 Hz	20-200+ Hz	300-1000 Hz	<0.2 Hz. Limited by safety considerations.
Line of sight issues?	Yes	Yes for some materials	No	Yes for some materials
Metal interference?	None	High	None	None
Ionizing radiation?	No	No	No	Yes

4.3     Assumptions for Speed of Sound in Tissue

Uncorrected speed of sound discrepancies in soft tissue can result in US target localization errors up to a few millimeters. Salter, et al. [76] found speed artifact errors of 0.7 mm per cm of fat traversed in experiments conducted in a phantom. To correct for such discrepancies, Fontanarosa, et al. [77] applied a density-based speed of sound correction algorithm to a set of prostate, liver, and breast images collected from volunteers. Algorithms applied corrections to the speed of sound based on tissue densities drawn from CT scans co-registered to the simulation US scans. Results indicated prostate, liver, and breast centroid shifts of 3.6, 6, and 1.3 mm, respectively, and liver volume changes of up to 9% when compared to uncorrected data. In later studies, a correction algorithm was applied to the scanning of a multi-modality US phantom through three different layers of liquid, with results showing differences between US and CT images smaller than the resolution of the CT scan (around 0.7 mm in the image plane) [78]. Additional studies by this group estimated errors in prostate location based on speed-of-sound aberration that agreed well with previously published discrepancies between US and CT scans, with corrections to prostate centroids averaging 3.1 mm [78]. With these discrepancies exceeding typical tissue tracking accuracies of US guidance systems previously discussed, results indicate a need to consider the speed of sound aberrations in estimating tumor location.

4.4     Imaging Performance During Beam Delivery

**Figure 5:**
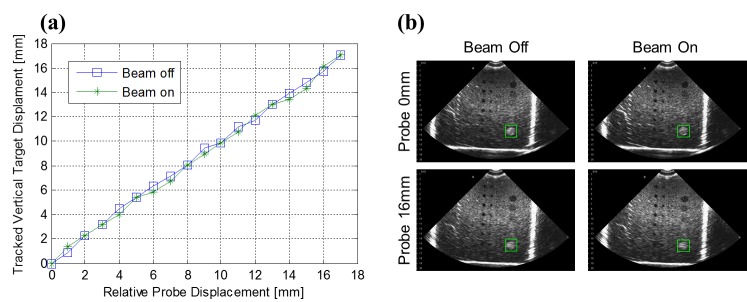
Comparison of US images and tracking performance with beam off and on, reproduced from Schlosser et al. Comparison of US images and tracking performance with beam off and on, reproduced from Schlosser et al. [58] (a) True object displacement versus computed displacement using cross-correlation. (b) Tracked circular object indicated within ultrasound images at two displacement values. The square indicates the location of the tracked object based on the manually selected target template.

Studies examining the effect of radiation on US image quality have produced dissimilar conclusions, although all have demonstrated the feasibility of US ultrasound to track anatomical motion in real-time. Hsu, et al. [79] used an Acuson 128/XP US scanner to image stationary and moving phantoms while operating an Elekta SL25 LINAC. The experiment found the US images to be affected by a periodic noise at a frequency identical to the pulsing frequency of the treatment machine; however, this noise was found to have minimal effects on the precision of the tracking algorithm. Schlosser, et al. [58] found no spatial or temporal interference patterns in US images of a phantom acquired with a 3.5 MHz Interson 2D single-element probe during LINAC beam operation. Additionally, no significant difference in tracking accuracy was found between beam-off and beam-on cases (Figure 5). US imaging interference from a LINAC likely depends on the specifics of the US imaging system being used; for example, modern systems with improved shielding may better reject radio-frequency interference from the LINAC.

4.5     Treatment Planning for Intrafractional Guidance

**Figure 6:**
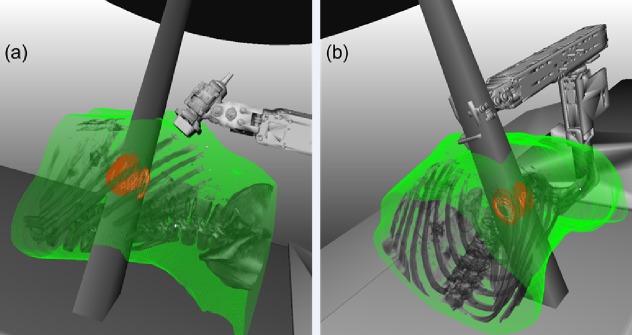
US imaging hardware configurations that (a) do not interfere and (b) interfere with certain treatment beams. Reproduced from Schlosser[63].

Intrafractional US imaging hardware (US probe, robot, and probe tracking sensor) in the treatment field may absorb radiation and alter the dose delivered to the patient. To minimize chances of beam interference with US hardware (Figure 6), our group is collaborating with researchers at the University of Lubeck to develop US probe placement assistance software. The software analyzes the patient’s CT data to suggest probe placements that achieve a clear acoustic window to the target while avoiding common beam delivery paths (Scheduled to be presented in 2015 at time of writing: Gong R, Bruder R, Schweikard A, Schlosser J, Hristov D: Augmented Reality System for Ultrasound Guided Radiation Therapy. In Computer Assisted Radiology and Surgery Conference. Barcelona, Spain; 2015). However, certain patient-specific anatomical configurations may preclude hardware placements that completely avoid beam delivery paths, and in these cases, intrafractional US hardware must be taken into account during the beam planning process. In general, this can be accomplished in two ways:

1. Avoid treatment beam positions that interfere with hardware.

2. Deliver radiation through the US image guidance hardware and account for hardware in the treatment planning process.

**Figure 7:**
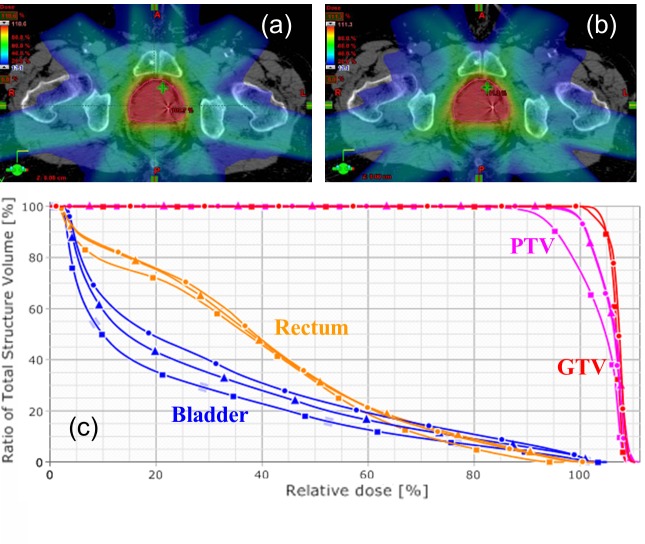
Seven-beam treatment plan comparison, reproduced from Schlosser et al. Seven-beam treatment plan comparison, reproduced from Schlosser et al. [58] (a) Axial dose distribution for a clinical prostate IMRT plan. (b) Axial dose distribution for a reoptimized IMRT plan with restricted beam angles to avoid U.S. probe and robot links. (c) Dose-volume histograms for the clinical IMRT plan (circles), reoptimized plan (triangles), and reoptimized plan with reduced margin (squares). Note that the IMRT plan with reduced margins underdoses original planning target volume but maintains gross tumor volume coverage and improves healthy tissue sparing.

Several groups have investigated the feasibility of strategy (1) by studying whether constraints on beam angles imposed by intrafractional US image guidance hardware affect the quality of treatment. Wu, et al. [81] found that avoiding the anterior-posterior beam in radiation planning, which would pass through a US probe placed in the transabdominal imaging position, resulted in a negligible effect of the transducer on dosage delivered. Schlosser, et al. [58] conducted a second feasibility study that compared a seven-beam clinical plan for a prostate IMRT patient with a seven-beam plan for the same patient that excluded a 90 degree anterior sector in order to avoid US guidance hardware in the transabdominal imaging position. No impactful difference between the plans was found (Figure 7). These two studies show that beam avoidance of US image guidance hardware is a feasible option for delivering prostate radiotherapy guided by transabdominal US imaging.

Zhong, et al. [82] examined the effect of probe orientation on liver SBRT plans that avoid an intrafractional US imaging probe. The study compared clinically accepted SBRT plans for 10 patients with liver cancer with two new plans generated for each patient that avoided irradiating the US probe. One of these two plans positioned the probe on the surface of the abdomen parallel to the patient’s longitudinal axis, and the other positioned the probe vertical to the longitudinal axis. Treatment plans could not be generated for two patients with superficially located tumors for either probe orientation. For the remaining eight patients, plans were successfully generated that did not show significant differences in dosage delivery metrics. With a treatment goal of delivering 37.5 Gy to the PTV in 3 fractions, the average dose delivered to 95% of the PTV was evaluated to be 38.63 ± 0.14 Gy for probe orientations parallel to the longitudinal axis; 38.48 ± 0.31 Gy for probe orientations vertical to the longitudinal axis; and 38.72 ± 0.14 Gy for clinical SBRT plans. The authors conclude that, excluding superficial lesions, US monitoring in real-time during liver SBRT is feasible.

For delivery of radiation through US guidance hardware (strategy (2) above), Bazalova, et al. [83] accounted for the presence of US probes by developing Monte Carlo models of two Philips US probes: the X6-1 3D/4D transducer and the C5-2 2D transducer. Models were built based on their respective TomoTherapy images, and mass densities were assigned based on an electron density calibration phantom. Beam attenuation due to the probes was then measured in a solid water phantom for a 6 MV and a 15 MV beam, and the results were compared to predictions based on the Monte Carlo model. The two methods corresponded well, with over 98% of measurement points passing the 3%/3 mm criteria for both probes and measurement depths. The extreme attenuation for probes in the upright position was found to be 25% and 31% for the C5-2 and the X6-1, respectively, for both 6 MV and 15 MV beams at a 10 cm depth. Results demonstrate the need for inclusion of a hardware model in beam treatment planning and indicate the feasibility of using Monte Carlo calculations to predict the dose delivered through traditional US probe hardware. To further mitigate potential dose interference from the US probe, our group is developing a novel radiolucent 3D/4D US image device that minimizes dose absorption through imaging hardware.

## Conclusions

Recent years have seen increasing research and commercial activity toward ultrasound-based image guidance systems for radiation therapy. The BAT, Sonarray, and Clarity systems for inter-fractional imaging demonstrated early success in deploying US for image guidance, but technical limitations stymied the widespread adoption. The commercially available Clarity Autoscan system has moved US into the realm of intrafractional imaging for a specific application – treatment of prostate lesions. Current research aims to build out the components necessary for an intrafractional US image guidance system that integrates with existing LINACs and has the capability to image a variety of abdominal and pelvic cancers. A survey of emerging research shows great progress towards generalized intrafractional US guidance with robotically controlled US probe manipulators, robust real-time tissue tracking algorithms, and techniques for incorporating image guidance hardware into the radiotherapy treatment plan. 
